# Self-Assembly of a [1 + 1] Ionic Hexagonal Macrocycle and Its Antiproliferative Activity

**DOI:** 10.3389/fchem.2018.00087

**Published:** 2018-04-03

**Authors:** Khushwant Singh, Ankit Gangrade, Sourav Bhowmick, Achintya Jana, Biman B. Mandal, Neeladri Das

**Affiliations:** ^1^Department of Chemistry, Indian Institute of Technology Patna, Bihta, India; ^2^Department of Biosciences and Bioengineering, Indian Institute of Technology Guwahati, Guwahati, India

**Keywords:** ionic metallamacrocycle, supramolecular assemblies, cytotoxicity, cancer cell lines, heterocyclic compounds

## Abstract

A unique irregular hexagon was self-assembled using an organic donor clip (bearing terminal pyridyl units) and a complementary organometallic acceptor clip. The resulting metallamacrocycle was characterized by multinuclear NMR, mass spectrometry, and elemental analyses. Molecular modeling confirmed hexagonal shaped cavity for this metallamacrocycle which is a unique example of a discrete hexagonal framework self-assembled from only two building blocks. Cytotoxicity of the Pt-based acceptor tecton and the self-assembled Pt^II^-based macrocycle was evaluated using three cancer cell lines and results were compared with cisplatin. Results confirmed a positive effect of the metallamacrocycle formation on cell growth inhibition.

## Introduction

Coordination driven self-assembly has been used conveniently in contemporary research for the construction of wide range of discrete supramolecular architectures (Chakrabarty et al., 2011; Cook et al., 2013b; Li et al., 2013; Schmidt et al., 2014; Schoedel and Zaworotko, 2014; Tanaka et al., 2014; Bhowmick et al., 2015a,b; Cook and Stang, 2015; Wang et al., 2016; Jana et al., 2017). In such complex frameworks, the building blocks (*aka* supramolecular tectons) are held together by using multiple ligand-metal coordination bonds (Lehn, 1995; Chakrabarty et al., 2011; Cook et al., 2013b; Li et al., 2013; Ward and Raithby, 2013; Han et al., 2014; Schmidt et al., 2014; Schoedel and Zaworotko, 2014; Tanaka et al., 2014; Bhowmick et al., 2015a,b; Cook and Stang, 2015; Johnson et al., 2015; Holloway et al., 2016, 2017; Wang et al., 2016; Jana et al., 2017). The dimensions of resultant abiotic supramolecules are guided by chemical information (stereo and geometric) contained in precursor moieties (Oliveri et al., 2008; Newkome and Shreiner, 2010; Inokuma et al., 2011; Cook et al., 2013b; Harris et al., 2013; Yoshizawa and Klosterman, 2014; Cook and Stang, 2015; Wang et al., 2015; Xu et al., 2016; Jana and Das, 2017). Intense research interest in understanding the complexity level of these artificial self-assembly processes is reflected in the quantum of reports published in this research domain (Barbara, 2001; Sauvage, 2001; Balzani et al., 2002; Beuerle, 2016; Fujita et al., 2016). Moreover, metal-mediated self-assembly studies are useful in understanding the fundamental principles of molecular self-organization in nature (Liu et al., 2007; Albertí et al., 2013; Chakraborty et al., 2013, 2015; Cook et al., 2013b; Galstyan et al., 2013; Saha et al., 2014; Sarkar et al., 2014; Chen et al., 2015; Cook and Stang, 2015; Li et al., 2015).

The library of discrete structures designed using the principles of coordination driven self-assembly (Pluth et al., 2009; Yoshizawa et al., 2009; Michito and Makoto, 2010; Thanasekaran et al., 2012; Cook et al., 2013b; Therrien, 2013; Young and Hay, 2013; Mishra and Gupta, 2014; Samanta and Mukherjee, 2014; Xu et al., 2014; Cook and Stang, 2015; Bhowmick et al., 2016) include two dimensional (2-D) structures such as metallamacrocycles (triangles, rectangles, pentagon, hexagon, square etc.) and three dimensional (3-D) frameworks (cages, boxes, barrels, prisms, Archimedean, and Platonic solid) (Stang and Olenyuk, 1997; Fujita et al., 2005; Takezawa and Shionoya, 2012; Cook et al., 2013b; Kishi et al., 2013; Chen et al., 2014; Liu et al., 2014; Lu et al., 2014; Cook and Stang, 2015; Manna et al., 2015; Zhang et al., 2017). Among the 2-D polygons, hexagonal frameworks are most interesting because such a shape is most abundantly observed in nature. Prominent examples of hexagonal motif observed in nature are honeycombs and graphite. Artificial self-assembly of hexagonal nanoscalar entities is challenging, as it requires convergence of several smaller components.

In recent years, an emerging application of these abiological supramolecules is to investigate their nature of interactions with biological systems (Wang and Lippard, 2005; Kelland, 2007; Wheate et al., 2010). These interactions include but are not limited to studies with cancer cells, DNA and proteins. Among purely inorganic complexes, Pt, Ru, and Au based ions are most popular (Mattsson et al., 2009; Ott, 2009; Berners-Price and Filipovska, 2011; Vajpayee et al., 2011, 2013; Lo et al., 2015; Ajibola et al., 2017). For example, Pt based cisplatin has been one of the most successful therapeutic agent in cancer treatment (Rosenberg et al., 1969; Vickers et al., 2004). However cisplatin has limitation (as a chemotherapeutic agent) such as uptake by healthy cells, resistance developed by target cancer cells, harmful side effects (such as nephrotoxicity, neurotoxicity and ototoxicity) and protein inactivation (Jung and Lippard, 2007; Yao et al., 2007; Wang and Guo, 2008; Todd and Lippard, 2009; Kaluderovic and Paschke, 2011; Barry and Sadler, 2013; Farrell, 2015). Considering these limitations of cisplatin's therapeutic use, synthetic organometallic and supramolecular entities are being explored as alternatives with potential application in treatment of cancer. These supramolecules are often constructed from organometallic precursor molecules. In this class of molecules, the metal ruthenium is clearly a leader (Yan et al., 1997; Therrien et al., 2008; Paul et al., 2012; Jo et al., 2017). Cook, Stang, and Chi have reviewed the biological interactions of metallacycles derived using coordination driven self-assembly protocol (Cook et al., 2013a). Majority of these are derived from Ru based precursors. It is obvious from this report that biochemical interactions of supramolecular frameworks bearing Pt(II) centers have not been explored, especially in the context of their potential as anticancer therapeutic agents.

This present work is in continuation of our research efforts to design unique supramolecular coordination complexes (SCCs) wherein we report synthesis of a discrete and nanoscalar hexagonal supramolecular complex using a new donor tecton. Furthermore, we have explored cytotoxicity of the new hexagonal SCC and its organometallic precursor. Additionally the results have been compared with cisplatin under similar conditions.

## Experimental section

### General details

All chemicals and anhydrous solvents used in this work were purchased from commercial sources and used without further purification. FTIR spectra were recorded in a PerkinElmer Spectrum 400 FT-IR spectrophotometer. ^1^H and ^31^P{^1^H} NMR spectra were recorded on Bruker 400 MHz spectrometer. Elemental analyses were carried out using an Elementar Vario Micro Cube elemental analyzer. ESI-MS analysis was performed using a Bruker Impact ESI-Q-TOF system. Theoretical calculations of PM6 semiempirical molecular orbital method were carried out with Gaussian 09. A549 (human lung cancer cell line), KB (human oral cancer cell line) and HaCaT (human skin keratinocyte cell line) were procured from National Centre for Cell Science (NCCS), Pune. MTT [(3-(4, 5-dimethylthiazol-2-yl)-2, 5-diphenyl tetrasodium bromide] was purchased from SigmaAldrich, USA. Ethidium homodimer-1 in 2 mL pbs, propidium iodide, Ribonuclease A were also purchased from SigmaAldrich.

### Synthesis of compound 5

2,6-bis((3-iodophenyl)ethynyl)pyrazine **3** (0.050g, 0.093 mmol), 4-ethynyl-pyridine **4** (0.019 g, 0.187 mmol), CuI (0.017 g, 0.009 mmol) and bis(-triphenylphosphine)palladium(II) dichloride (6.52 mg, 0.009 mmol) were charged in a 50 ml Schlenk flask in the glove box. Subsequently, 10 ml dry THF and freshly distilled and degassed triethylamine (0.5 ml, 0.372 mmol) were added under nitrogen. The reaction mixture was stirred overnight at room temperature. After overnight stirring, the reaction mixture was filtered through a bed of celite. The filtrate obtained was evaporated to dryness on a rotary evaporator to obtain a crude product which was purified by column chromatography on neutral alumina by eluting with 35% ethyl acetate in hexane to isolate the desired product (**5**) as off white solid.

Yield: 0.037 g, 81%, mp 198–202°C; ^1^H NMR (400 MHz, CDCl_3_): δ 8.67 (s, 2H, Ar-H), 8.62–8.61 (dd, *J* = 6 Hz, 2H, Ar-H), 7.81–7.80 (m, 1H, Ar-H), 7.64–7.575 (m, 2H, Ar-H), 7.43–7.37 (m, 3H, Ar-H).^13^C{^1^H} NMR (CDCl_3_, 100 MHz): δ 149.8, 145.7, 139.5, 135.3, 132.9, 132.7, 130.9, 128.9, 125.5, 122.8, 121.9, 92.4, 92.3, 87.6, 86.1. IR (ATR): 3,048, 2,918, 2,849, 2,210, 1,687, 1,585, 1,503, 1,404, 1,279, 1,203, 1,154, 987, 887, 796, 671 cm^−1^. Anal. Calcd. For C_34_H_18_N_4_: C, 84.63; H, 3.76; N, 11.61. Found: C, 84.72; H, 3.84; N, 11.68.HRMS (ESI, *m/z*): Calculated for C_34_H_18_N_4_ ([M + H]^+^): 483.16; Found: 483.16.

### Synthesis of macrocycle 7

To the solution of **6** (30 mg, 0.020 mmol) in chloroform (4 mL) was added two equivalents of AgNO_3_ (7.06 mg, 0.040 mmol) in one portion, and the reaction mixture was stirred overnight in the absence of light at room temperature. The precipitated AgI was filtered off over a bed of Celite, and the filtrate was collected as a yellow colored solution. Subsequently, a methanolic solution of the donor tecton **5** (0.02 mmol, 0.5 mL) was added drop wise to the aforementioned filtrate with continuous stirring. The reaction mixture was stirred for 15 h at room temperature. Solvents were removed by rotary evaporator and the product obtained which was washed several times with *n*-pentane to obtain a solid that was finally dried in a vacuum. The macrocycle **7** was recrystallized as an off white microcrystalline solid by slow vapor diffusion of diethyl ether in its corresponding concentrated chloroform-methanol solution.

#### Macrocycle 7

Yield: 34 mg, 92%; ^1^H NMR (400 MHz, CDCl_3_): δ 8.89–8.87 (dd, *J* = 8 Hz, 4H, Ar-H), 8.68 (s, 2H, Ar-H), 8.67 (s, 2H, Ar-H), 8.00–7.99 (m, 4H, Ar-H), 7.89–7.87 (dd, *J* = 8 Hz, 4H, Ar-H), 7.68–7.64 (m, 4H, Ar-H), 7.47–7.45 (m, 4H, Ar-H), 7.33–7.32 (m, 4H, Ar-H), 1.86–1.83 (m, 8H, -CH_2_-), 1.26–1.20 (m, 12H, -CH_3_). ^31^P NMR (162 MHz, CDCl_3_): δ 15.70 (^1^*J*_PPt_ = 1,162 Hz). FTIR (ATR): 2,972, 2,922, 2,216, 2,116, 1,710, 1,602, 1,511, 1,469, 1,329, 1,145, 1,034, 888, 762, 683 cm^−1^. Anal. Calcd. for C_82_H_88_N_8_O_6_P_4_Pt_2_: C, 54.85; H, 4.94; N, 6.24. Found: C, 54.93; H, 4.98; N, 6.28. ESI-MS *m/z* found: [**7**−2NO_3_]^2+^ = 835.76.

### Preparation of stock solutions

Organometallic complex **6** and self-assembled macrocycle **7** were solubilized into cell culture grade dimethyl sulfoxide to make 10 mM stock solution. However, cisplatin was solubilized into saline (0.9% sodium chloride) to make 1 mM stock solution. Aliquots of the stock solutions were taken and preserved in −20°C till further use.

### *In vitro* cytotoxicity

The cytotoxicity of all the compounds (**6, 7** and cisplatin) was assessed using MTT assay against A549, KB, and HaCaT cell lines. First, **6** and **7** were solubilized in DMSO to prepare stock solutions (concentration 10 mM). Subsequently, stock solutions were diluted 1,000 times in cell culture media for the treatment. The compounds were then tested at varying concentration range starts from 2.5 to 30 μM. For the assay, all the cells were trypsinized and counted using hemocytometer. Approximately 5 × 10^3^ cells were seeded into each well of a 96 well plate and allowed to adhere for 24 h. Post adherence the cells were treated with pre-determined concentration (2.5, 5, 10, 15, 20, 25, and 30 μM) of all the compounds and incubated for 48 h. The media was then decanted, and cells were washed with phosphate buffer saline (PBS), pH 7.4. 100 μl of 10% MTT (5mg/ml stock) prepared in FBS free media was then added to each well and incubated at 37°C for 4 h. The formed formazan crystals were solubilized in DMSO and absorbance was taken at 570 nm using the Multiplate reader (Tecan Infinite Pro, Switzerland). The percentage viability was calculated by normalizing the absorbance value of test sample with anuntreated control, and the seven-point dose-dependent curve was plotted to determine the IC_50_ (IC_50_ value is the concentration of compound at which 50% of cells are viable) value of each compound.

### Live/dead assay

The viability of cells was visualized under a fluorescence microscope using live/dead solution. The cells were seeded in a 96 well plate as mentioned earlier. Post adherence cells were treated for 48 h with **6-7** at 25 μM concentrations and cisplatin at 10 μM concentration. The media was decanted and after PBS (Phosphate-buffered saline) wash, cells were incubated with 100 μl working live/dead solution (1 μL of 4 mM Calcein AM and 4 μL of 2 mM ethidium homodimer-1 in 2 mL PBS) for 10 min at 37°C. The live cells fluoresce green (Excitation: 495 nm, Emission: 516 nm) due to Calcein-AM uptake however dead cells fluoresce red (Excitation: 528 nm, Emission: 617 nm) due to Ethidium homodimer-1 uptake.

## Results and discussion

### Synthesis and characterization of the organic donor clip (5)

**Scheme 1:**
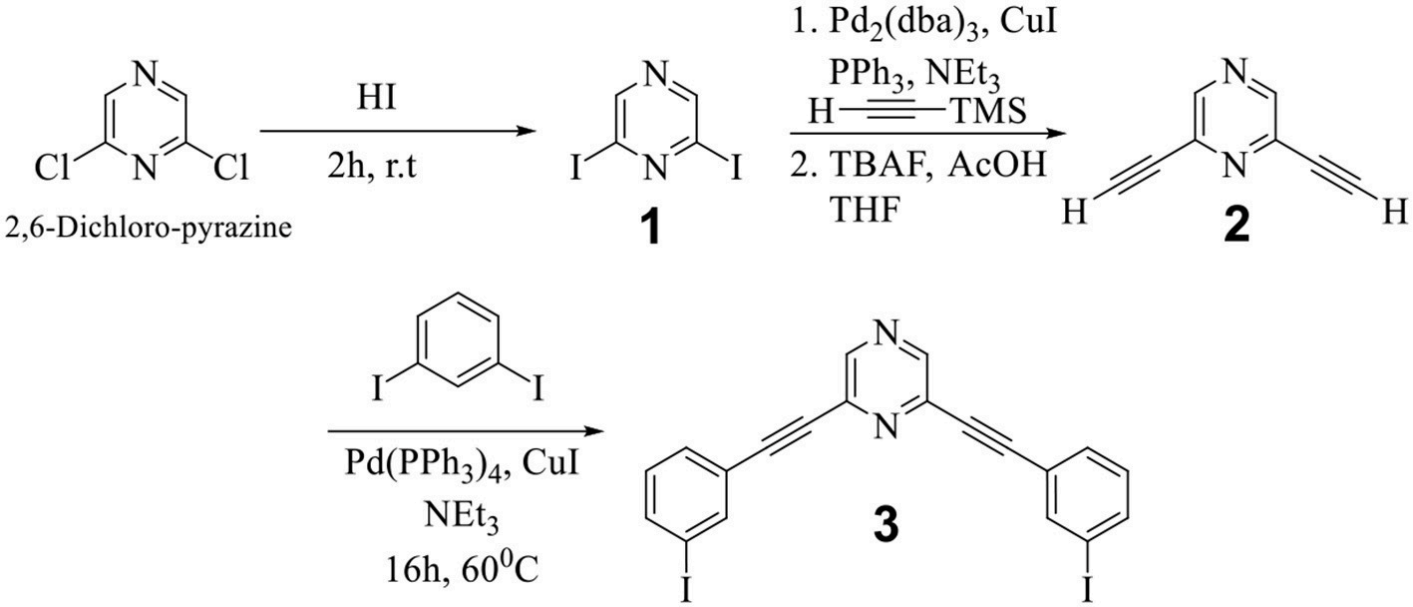
Synthesis of 2,6-bis((3-iodophenyl)ethynyl)pyrazine (**3**).

Commercially available 2,6-dichloropyrazine is the synthetic precursor for both donor and acceptor tectons employed herein for the self-assembly reaction. In the first step, 2,6-diiodopyrazine (**1**) was obtained from 2,6-dichloropyrazine, which was further reacted in two steps to yield 2,6-bis((3-iodophenyl)ethynyl)pyrazine (**3**) via the formation of 2,6-diethynylpyrazine (**2**) (Scheme 1) (Bhowmick et al., 2017).

**Scheme 2:**
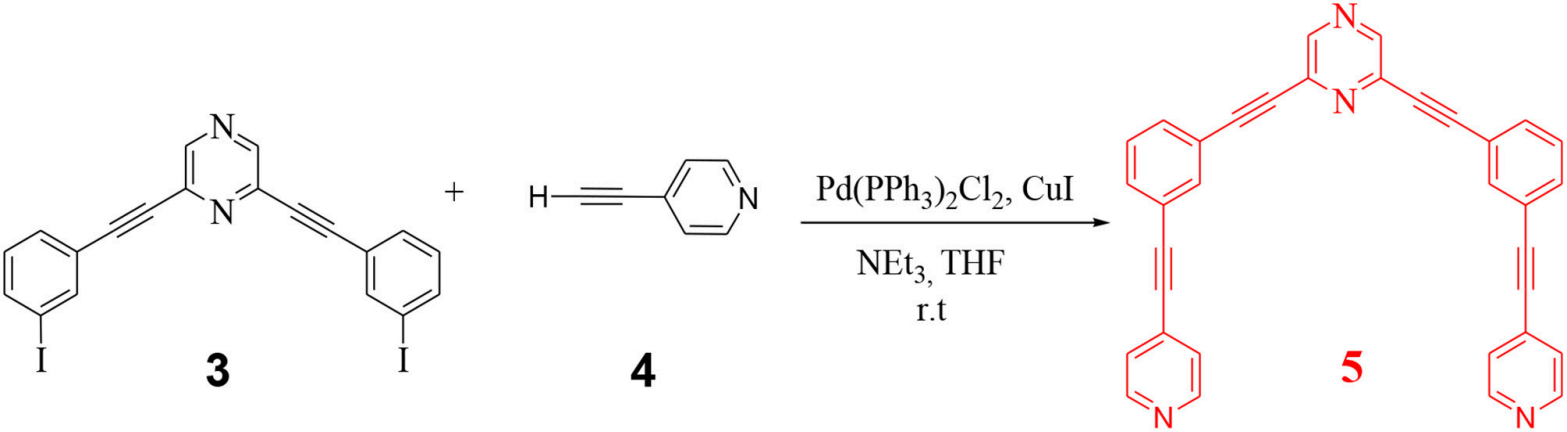
Synthesis of organic donor clip (**5**).

In the final step, **3** was further reacted with 4-ethynyl pyridine (**4**) to yield the desired organic donor clip (**5**) as depicted in Scheme 2. 2,6-bis((4-ethynylpyridyl)ethynyl)pyrazine) (**5**) was obtained as a white colored compound (81% isolated yield). **5** is stable in air/moisture and has high solubility in common organic solvents. **5** was fully characterized by FT-IR and NMR spectroscopy, mass spectrometry and elemental analyses.

In the ^1^H NMR of **5** (Figure S1), signal appearing at δ = 8.62 ppm and δ = 7.64 ppm corresponds to the α and β pyridyl protons respectively. As expected, signals corresponding to these protons appear as doublets. All characteristic peaks corresponding to pyrazine, phenyl and pyridyl units of **5** were observed in ^13^C {^1^H} NMR spectrum (Figure S2).

### Application of 5 as a donor tecton toward self-assembly of a supramolecular hexagon

Coordination driven self-assembly reactions have been employed for the construction of complex yet discrete metallomacrocycles. Among various shapes reported in the literature, hexagonal macrocycles are especially fascinating because of nature's preference for hexagon motif (honeycomb, graphite, snowflakes, etc.) (Pucci et al., 2000). Consequently, there is interest in mimicking nature in laboratory in the context of design and synthesis of macrocycles with hexagonal cavity. The most commonly reported methodologies involve self-organization of 12 (twelve) or 6 (six) tectons, thereby generating hexagonal assemblies that are referred to as [6 + 6] or [3 + 3] hexagonal metallacycles respectively (Cook and Stang, 2015). These syntheses are challenging as they require self-assembly of a large number of molecular components in a reaction that is entropically unfavorable. More recently, we have reported design and synthesis of hexagonal polygons requiring self-assembly of two donor and two acceptor tectons thereby yielding [2 + 2] hexagons. In this report, we have chosen donor and acceptor in such a manner that construction of a hexagonal macrocycle requires only one donor and one acceptor tecton. Thus the anticipated framework will be a unique [1 + 1] hexagonal macrocycle requiring the least number of self-assembling molecular tectons for its construction.

**Scheme 3:**
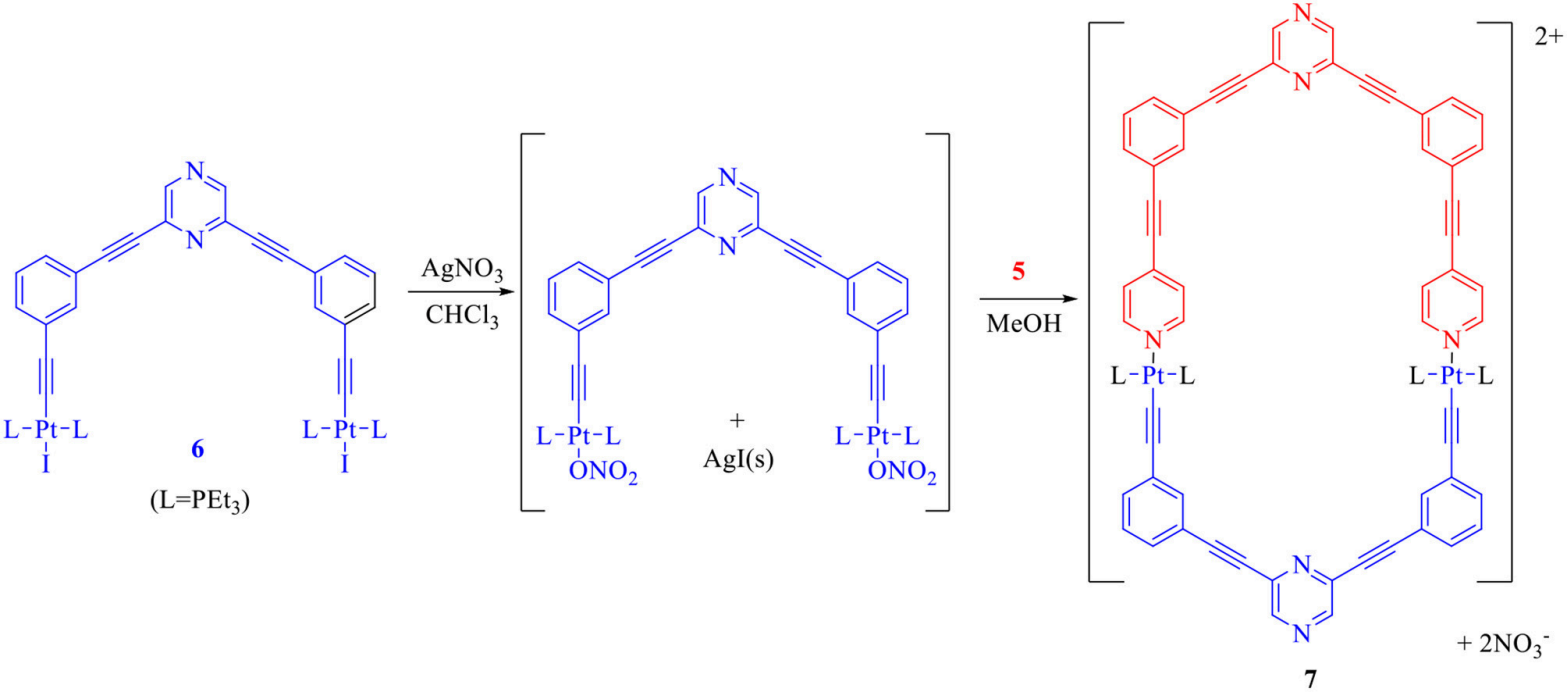
Design and self-assembly of a hexagonal macrocycle (**7**) by employing only two molecular tectons.

In order to perform the above mentioned self-assembly reaction, first the acceptor tecton (**6**) was reacted with two equivalents of AgNO_3_ in CHCl_3_ to yield the corresponding dinitrate derivative which was further reacted with the donor tecton (**5**) (methanol solution) in 1:1 stiochiometric ratio at room temperature (Scheme 3). A solid product was obtained by evaporating the solvents. Subsequent washing (with n-pentane) and recrystallization yielded the desired product (**7**) as an off-white microcrystalline solid (yield > 90%) that was soluble in organic solvents.

**Figure 1:**
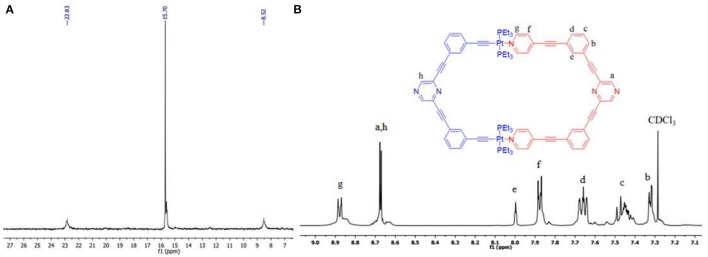
**(A)** ^31^P{^1^H} and **(B)** ^1^H NMR (partial) spectra of **7**. Lower case letters represent signals/peaks in proton NMR corresponding to different protons.

The self-assembled product (**7**) was subjected to various characterization techniques to confirm its purity and composition. In ^31^P{^1^H} NMR spectrum (Figure 1A) a sharp singlet (δ = 15.70 ppm), accompanied with a pair of ^195^Pt satellite peaks (^1^*J*_PPt_ = 1,162 Hz), indicated that **7** has a highly symmetrical structure, wherein all phosphorous nuclei are chemically equivalent. The ^1^H NMR spectrum of **7** also suggested incorporation of both tectons (**5** and **6**) in it (Figure 1B and Figures S3, S4). In ^1^H NMR spectrum of **7** (Figure 1B), two peaks appearing at 8.67 and 8.68 ppm were assigned to the protons of pyrazine moieties of donor (**5**) and acceptor clip (**6**) respectively. Four sets of signals at 8.00–7.99, 7.68–7.64, 7.49–7.45, 7.33–7.32 ppm are due to protons in phenyl ring. Two sets of signals at 8.89–8.87, 7.89–7.87 ppm are due to protons of the pyridyl ring. The ethyl protons of PEt_3_ groups (attached to Pt^II^ center) are observed in the range 1.86–1.20 ppm spectrum (Figure S3).

The formation of **7** via coordination of **5** (donor tecton) with **6** (acceptor tecton) was also evident from the observed downfield shift (Δδ ~ 0.25 ppm) of both α and β pyridyl protons present in the terminal pyridine moieties (Figure S5). This has been attributed to the decrease in electron density in pyridine rings of the donor (**5**) due to their coordination (via lone pair on nitrogen) with Pt^II^ metal centers of the acceptor tecton (**6**).

**Figure 2:**
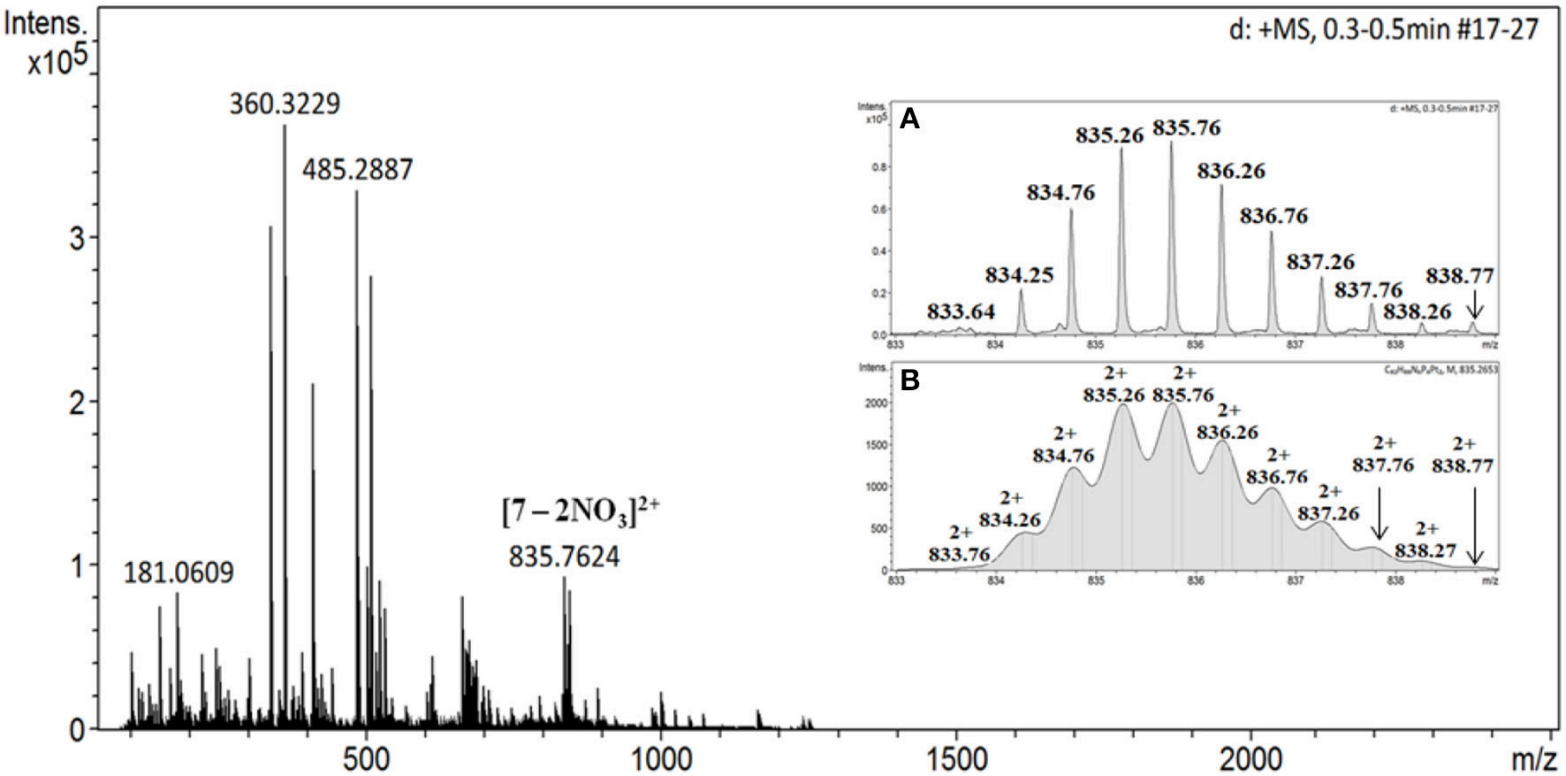
ESI-MS data of the macrocycle **7**; inset **(A)** experimental spectrum and **(B)** theoretical isotopic distribution pattern of the fragment [**7**-2NO_3_]^2+^.

Furthermore, the purity of product **7** was confirmed by ^1^H DOSY NMR spectroscopy (Figure S6), wherein a single trace spectrum indicated the formation of a single product. This result also confirms absence of other macrocyclic species or oligomers as byproduct and exclusive formation of discrete macrocyclic species. Thus NMR analyses confirm purity of the **7** and indicated formation of single, highly symmetrical species. Sharp peaks in ^1^H and ^31^P NMR are also clear indications of presence of discrete species and absence of any oligomers. Mass spectrometry was subsequently used to confirm the proposed composition of **7**. The ESI-TOF-MS spectrum of **7** indicated formation of desired [1 + 1] molecular ensembles (Figure 2). The ESI-TOF-MS spectrum of **7** showed a signal corresponding to the consecutive loss of two nitrate counter anions from the expected [1 + 1] macrocycle at m/z = 835.76 [**7**-2NO_3_]^2+^. The isotopic resolution of this peak was in excellent agreement with the theoretically predicted isotopic distribution pattern of [**7**-2NO_3_]^2+^ (Figures 2A,B and Figure S8). Thus mass spectrometric analysis of **7** confirmed the formation of a discrete [1 + 1] self-assembled macrocycle.

**Figure 3:**
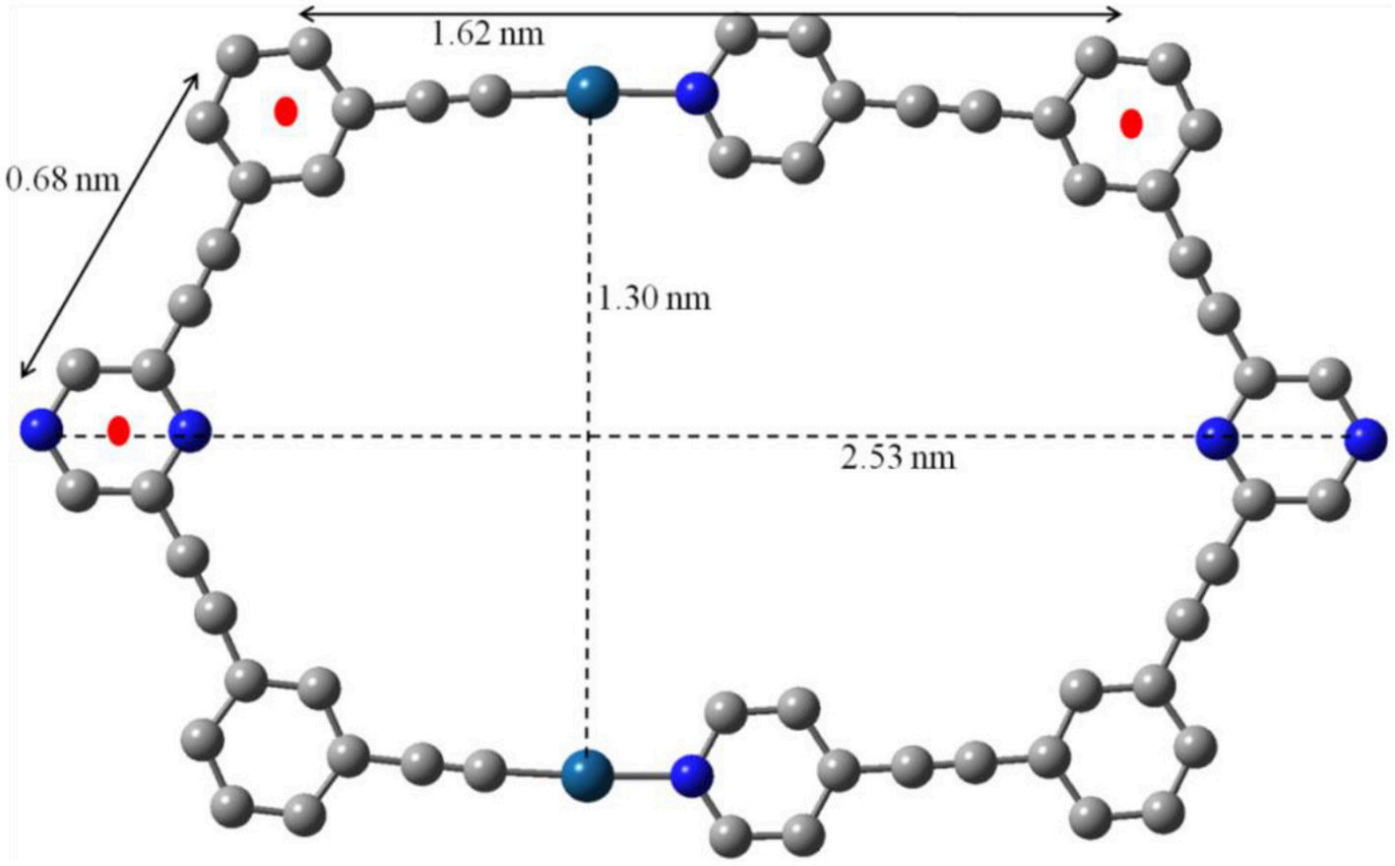
Simulated ball and stick molecular model optimized by PM6 semiempirical molecular orbital methods of macrocycle **7** (Color code: light gray, C; dark cyan, Pt; blue, N. PEt_3_ and Hare omitted for clarity).

All attempts to grow X-ray quality single crystals of **7** were unsuccessful. In such a scenario, molecular modeling (using PM6 semiempirical MO method) (Stewart, 2007) was employed to obtain useful structural information for **7**. The energy minimized structure of **7** (Figure 3 and Figure S7) confirms the formation of a macrocycle with hexagonal cavity. The distance between the two exocyclic nitrogen atoms of pyrazine rings was 2.53 nm. The distance between the two platinum centers was found to be 1.30 nm. The lengths of sides of hexagon were found to be 1.62 and 0.68 nm. Thus **7** may be described as an irregular hexagon since all the sides are not of equal length even though the polygonal framework is equiangular. A slight distortion from the square geometry was observed at the two Pt(II) centers.

### Cytotoxicity (*in vitro*) assessment and estimation of IC_50_

Till date, platinum based molecules have been used most frequently as anticancer therapeutic agents. Cisplatin is undoubtedly the most popular anticancer drug bearing Pt(II). In the first step of its mechanism of action (as anticancer drug), the chlorides of cisplatin are substituted/exchanged by hydroxide ions or water. The resulting diaquo species subsequently bind with nucleophilic sites present in RNA or DNA. Thus cytotoxicity of cisplatin stems from the fact that Pt-Cl bonds are unstable under physiological conditions relative to the new Pt-N bonds that are formed due to adduct formation with nucleobases. Also it is known that Pt-N bonds are essentially irreversible under the same physiological conditions (Farrell, 1999). Moreover, in the context of platinum based complexes as anticancer drugs, it is well known that cisplatin is quite potent while transplatin is inactive. This is a consequence of the difference in the stereospecificity of the labile Pt-Cl bonds in these two isomers. **6** and **7** are quite different from cisplatin in this aspect as these species don't have two labile (halide/hydroxo) groups in cis orientation to facilitate adduct (bifunctional) formation with DNA (as observed with cisplatin). It was therefore our curiosity to study the cytotoxicity of Pt(II) based compounds (**6** and **7**) reported herein that don't posses two cis-oriented labile groups unlike cisplatin.

**Figure 4:**
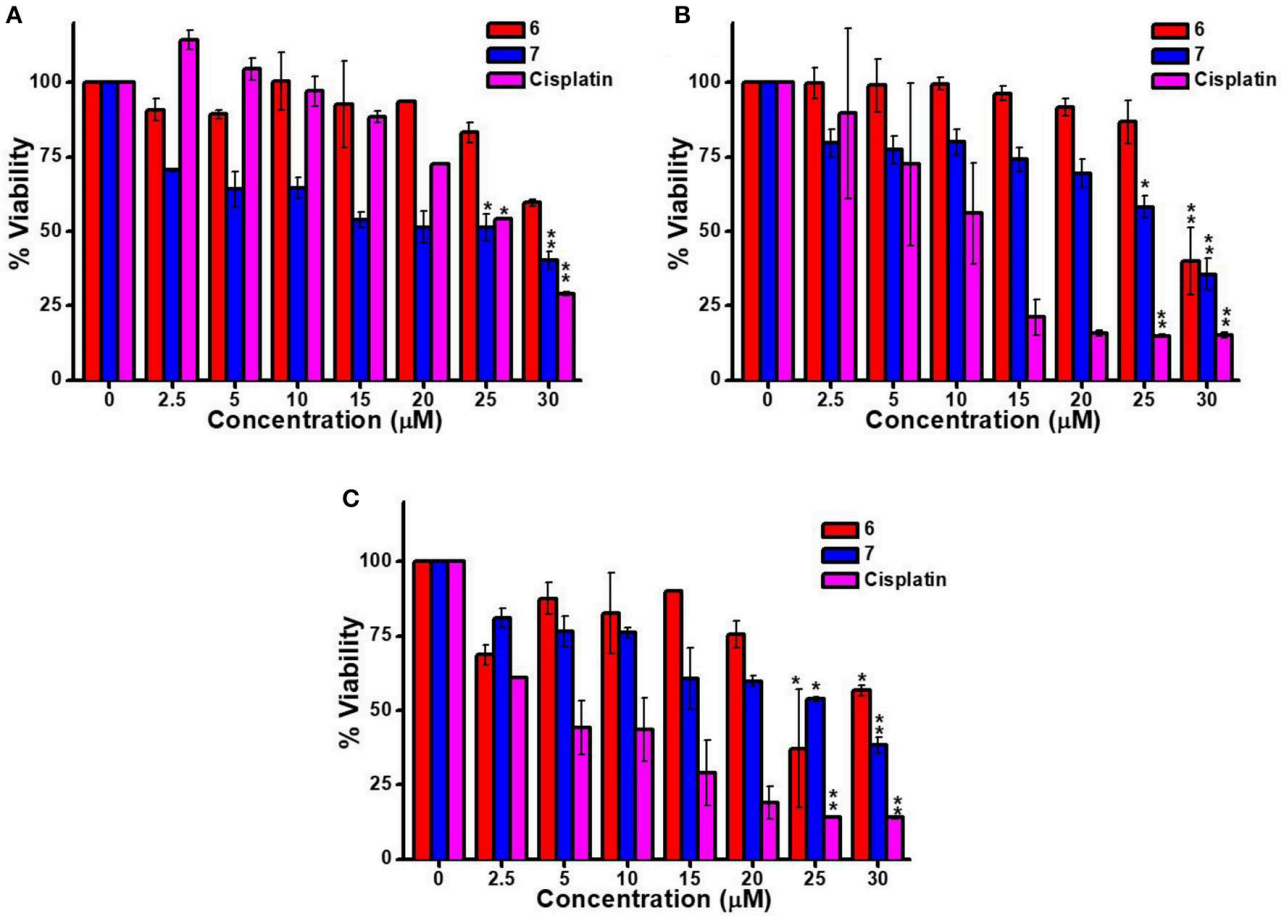
*In vitro* cytotoxicity of **6**, **7** and cisplatin with predetermined dose range against **(A)** A549 **(B)** KB, and **(C)** HaCaT cell lines for 48 h using MTT assay. Data points represent mean ± SD for three independent experiments. ^*^p < 0.05 and ^**^p < 0.01 represent a significant decrease in the viability of cells at 25 μM compounds treated group compared to the untreated group.

The cytotoxicity of platinum containing **6** and **7** were examined against three cell lines KB (human oral cancer), A549 (human lung cancer) and HaCaT (human skin keratinocyte) using MTT assay. Cisplatin was used as a positive control (Wang and Lippard, 2005). Toxicity of all the compounds was observed to be dose and cell dependent. The IC_50_ value is the inhibitory concentration of a compound at which 50% cells are dead. A seven-point dose-dependent plot (Figure 4) was recorded for each compound against each cell line for measurement of IC_50_ concentration. **7** exhibited higher toxicity (relatively lesser IC_50_ value) relative to **6** against A549 and KB cancer cells. However, for HaCaT cells, both **6** and **7** exhibited comparable IC_50_ value (Table 1).

**Table 1:** IC_50_ concentration (μM) after 48 h treatment against different cell lines.

	**IC**_**50**_**/**μ**M**		
	**A549 cells**	**KB cells**	**HaCaT cells**
Cisplatin	25.0 ± 2.0	11.0 ± 0.3	6.0 ± 3.0
**6**	>30.0	>30.0	23.0 ± 4.0
**7**	20.0 ± 0.4	26.0 ± 0.4	26.0 ± 0.2

From the IC_50_ values listed in Table 1, it is clear that compounds **6** and **7** have lesser toxicty relatively to cisplatin. This was anticipated considering the absence of two cis-oriented labile groups in **6** and **7**. In other words, the general higher activity of cisplatin over other **6** and **7** is due to the presence of cis-oriented more labile Pt-Cl bonds in cisplatin that tend to hydrolyze easily under physiological conditions to facilitate binding with DNA/RNA. On the other hand, the Pt^II^-based compounds in this report (**6** and **7**) have Pt-N bonds that tend to be relatively stable under such conditions.

It is however noteworthy that though **6** and **7** don't have labile groups in cis orientation to facilitate adduct (bifunctional) formation with DNA, these species show reasonable cytotoxic effect (Table 1). More interestingly, the macrocycle (**7**) exhibited superior cytotoxic effect when compared with cisplatin for A549 (human liver cancer) cells. Additionally, it was observed that upon self-assembly and formation of the macrocycle **7**, the resulting supramolecular framwork exhibited lower IC_50_ value relative to that observed for the acceptor tecton (**6**) in case of A549 and KB carcinoma cell. For, HaCaT cell, the IC_50_ values are comparable for both **6** and **7**. Considering the literature reported stability of Pt-N bonds under physiological conditions (Farrell, 1999), it may be assumed that macrocycle **7** remains intact under these conditions.

**Figure 5:**
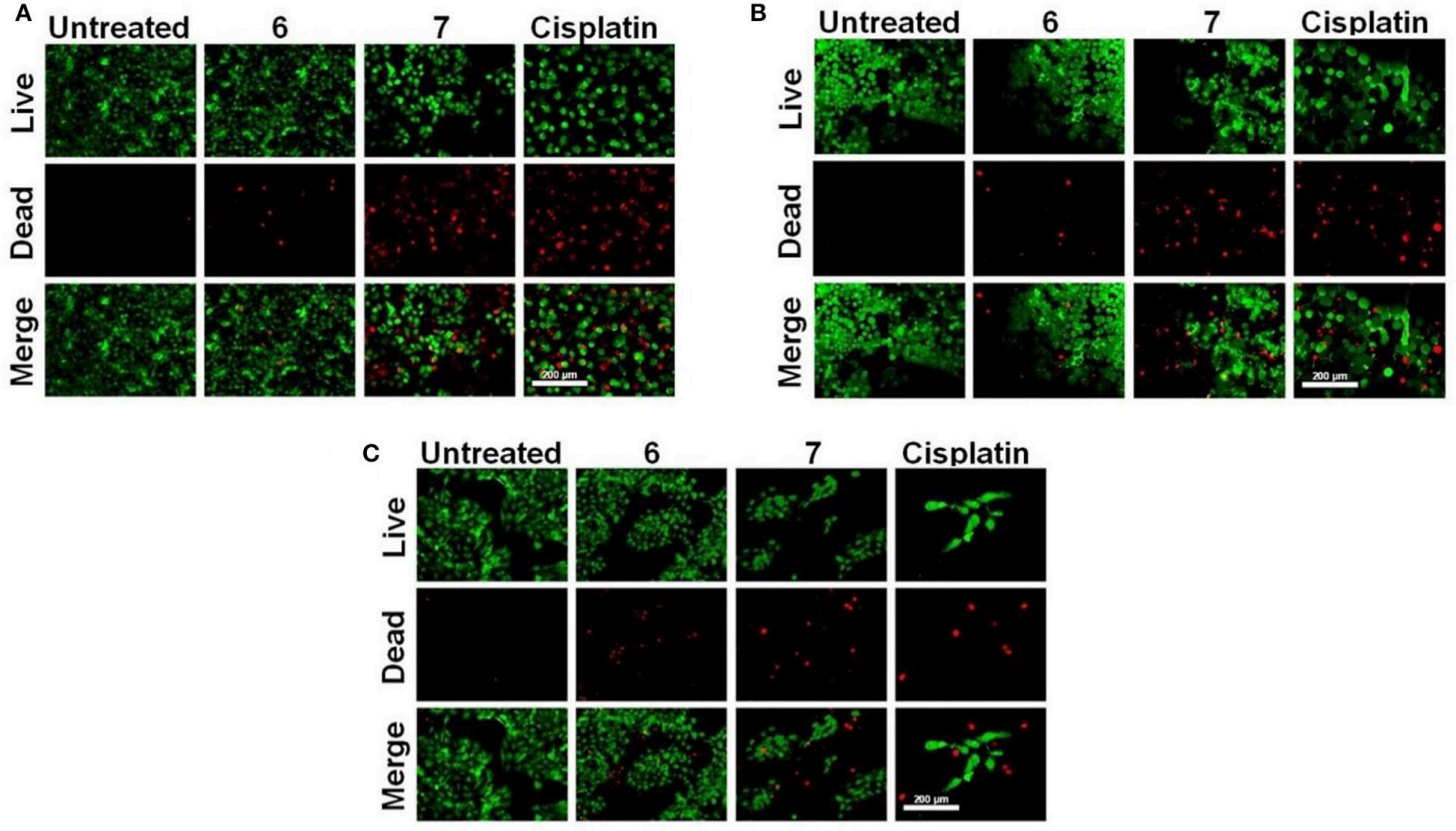
Live/dead cell staining. **(A)** A549, **(B)** KB, and **(C)** HaCaT cells were treated with 25 μM **6**, **7** and 10 μM cisplatin for 48 h. Post-treatment cells were stained with alive/dead stain solution, and the images were captured with a fluorescence microscope (scale bar – 200 μm).

Subsequently, the effect of Pt-based compounds (**6** and **7**) on cell viability and attachment was studied and results compared with cisplatin. Cancer cells (KB, A549, and HaCaT) were treated with compounds (**6**, **7** and cisplatin) and these were stained with fluorescent dyes (Calcein AM and Ethidium homodimer-1). In these fluorescent imaging studies, cancer cells were treated with 25 μM **6**-**7** and 10 μM cisplatin for 48 h. Post-treatment, cells were stained with a live/dead stainsolution. Fluorescent images are shown in Figure 5, wherein live cells (green color) were firmly attached to tissue culture plate (TCP) surface relative to dead cells (red color). Variable dead cell population was observed in each case due to either their loose attachment to TCP or difference in their respective IC_50_. These results suggest that **6** and **7** indeed demonstrate cancer cell growth inhibition to an extent that is comparable cisplatin (a widely used Pt-drug for treatment on various forms of cancer).

## Conclusion

In conclusion, we have reported a new donor tecton (**5**) with two pendant pyridine rings that is derived from commercially available 2,6-dichloropyrazine. **5** is a new ditopic donor tecton with 0° angular orientation of the two pyridine rings. Self-assembly reaction of **5** with a complementary ditopic acceptor tecton (**6**) resulted in the formation of a single product (**7**). NMR and elemental analyses confirmed its purity while mass spectrometry (ESI-MS) data supported the formation of a [1 + 1] supramolecular species. PM6 molecular modeling suggested formation of a macrocycle with hexagonal cavity. The product (**7**) is a unique example in literature wherein a nanoscalar, discrete and cationic hexagonal framework has been synthesized using only two tectons—one unit of donor and one unit of acceptor. Previous report of all Pt(II)-based hexagonal macrocycles utilized four, six or twelve tectons resulting in highly charged species. Furthermore the Pt^II^-based species (**6** and **7**) were subjected to cytotoxicity studies using three different cancer cell lines, and the results were compared with cisplatin. Although **6** and **7** are less potent than cisplatin, it was observed that formation of the metallamacrocycle (**7**) improved cytotoxicity in case of treatment with A549 and KB cancer cells. This is in spite of the fact that acceptor tecton (**6**) contains labile Pt-I bonds while macrocycle **7** contains platinum bonds to nitrogen that are essentially irreversible under physiological conditions (Farrell, 1999). Interestingly, the supramolecular ensemble (**7**) showed better A549 cell growth inhibitory effect than cisplatin. The cytotoxic behavior of **6** and **7** may be attributed to their non-covalent interaction with DNA. These results will form the basis of further research on the mechanism of cell-killing action of these Pt-based species which are stereo-chemically quite different from cisplatin. Nevertheless this report is a unique example, wherein a supramolecular hexagonal framework with only two Pt(II) centers has been self-assembled and it potential application in anticancer therapy has studied. Development of similar Pt(II)-based self-assembled structures as therapeutic agents for malignant cells is currently being explored in our laboratory.

## Author contributions

ND conceived the research and supervised the experimental work related to synthesis and characterization of organic/organometallic molecules reported herein. KS synthesized all new compounds reported in this manuscript. AJ optimized the energy-minimized geometry of the metallacycles **7**. SB assisted in the synthesis of some of the literature reported pyrazine precursors. AG carried out the experiments related to evaluation of biological activity of **6, 7** and cisplatin reported herein and these biological studies were supervised by BM. All authors have contributed to interpretation of results, compiled the manuscript and have approved the final manuscript.

### Conflict of interest statement

The authors declare that the research was conducted in the absence of any commercial or financial relationships that could be construed as a potential conflict of interest.

## Supplementary material

^1^H and ^13^C{^1^H} NMR spectra of **5**, ^1^H, ^31^P{^1^H} and ^1^H DOSY NMR spectra of the metallamacrocycle (**7**), comparison ^1^H NMR spectra of donor ligand **5** and macrocycle **7** and **s**pace-filled model of macrocycle **7** optimized by PM6 semiempirical molecular orbital method.
