# Supplementary material for: Self-Assembly of a [1 + 1] Ionic Hexagonal Macrocycle and Its Antiproliferative Activity
Source: Front Chem. 2018 Apr 3;6:87. doi: 10.3389/fchem.2018.00087 (PMC5891631; doi:10.3389/fchem.2018.00087)

Self-Assembly of a [1+1] Ionic Hexagonal Macrocycle and its Antiproliferative Activity

Khushwant Singh,[1] Ankit Gangrade,[2] Sourav Bhowmick,[1] Achintya Jana,[1] Biman B. Mandal,[2]* and Neeladri Das[1]*

[1]*Department of Chemistry, Indian Institute of Technology Patna, Bihta 801 106, Bihar, India*
[2]*Department of Biosciences and Bioengineering, Indian Institute of Technology Guwahati, Guwahati 781 039, Assam, India*

Table of contents

1. ^{1}H and ^{13}C NMR spectra of compound **5** S2
2. ^{1}H, ^{31}P and ^{1}H DOSY NMR spectra of compound **7** S3-4
3. Comparison ^{1}H NMR spectra of donor ligand **5** and macrocycle **7** S4
4. Space-filled model of macrocycle **7** optimized by PM6 semiempirical molecular orbital method. S5
5. ESI-MS data of the macrocycle **7**; inset (a) experimental spectra and (b) theoretical isotopic distribution pattern of the fragment [**7**-2NO_3]$^{2+}$. S6

Figure S1: ^{1}H NMR spectra of compound **5**

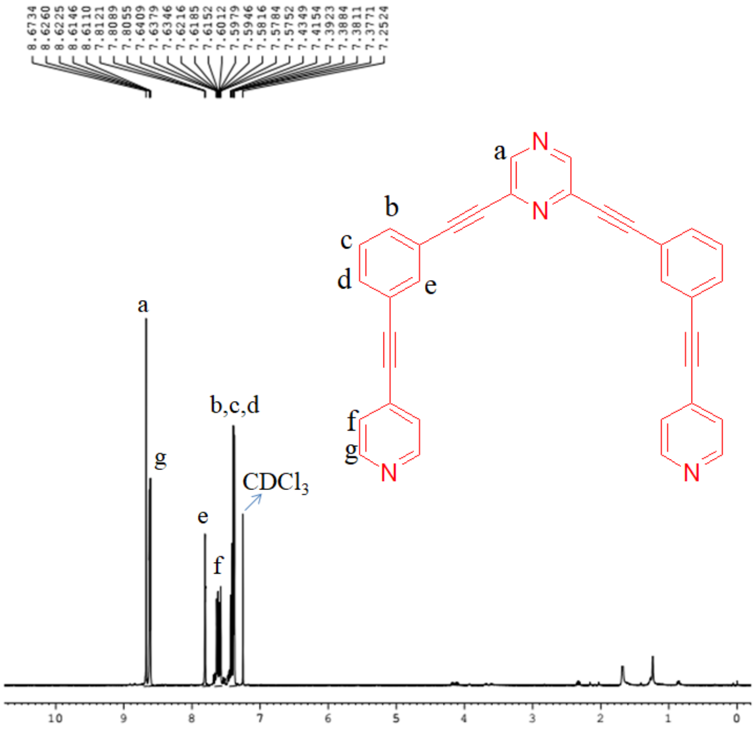

Figure S2: ^{13}C NMR spectra of compound **5**

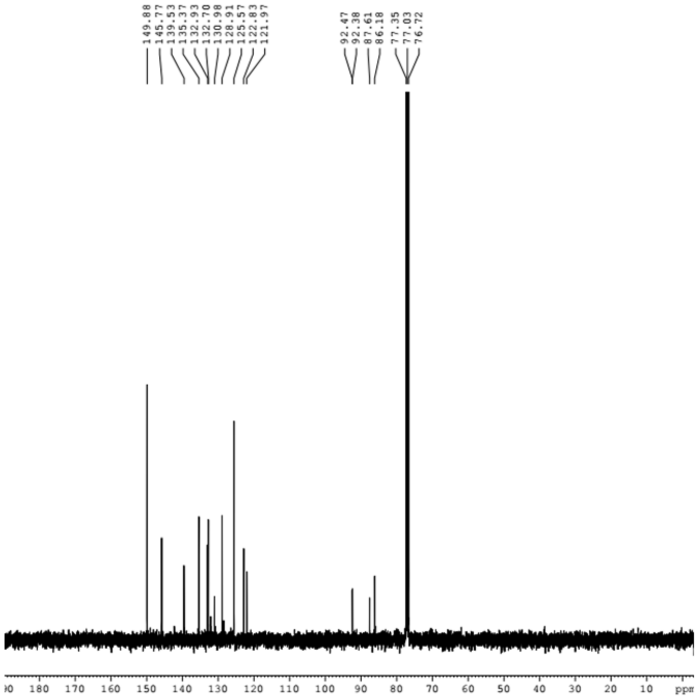

Figure S3: ^{1}H NMR spectra of macrocycle **7**

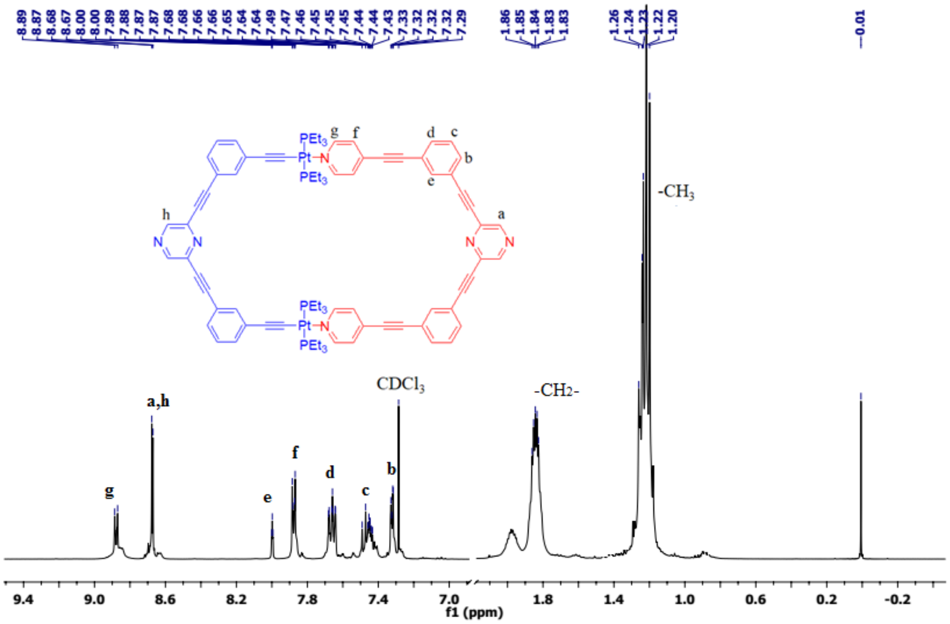

Figure S4: ^{31}P NMR spectra of macrocycle **7**

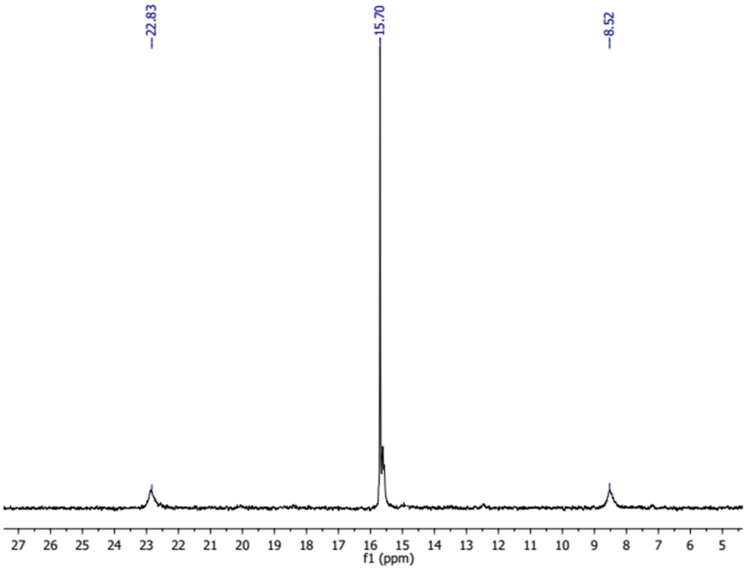

Figure S5: Comparison ^{1}H NMR spectra of (a) donor ligand **5** and (b) macrocycle **7** to confirm coordination driven self-assembly between **5** and **6**.

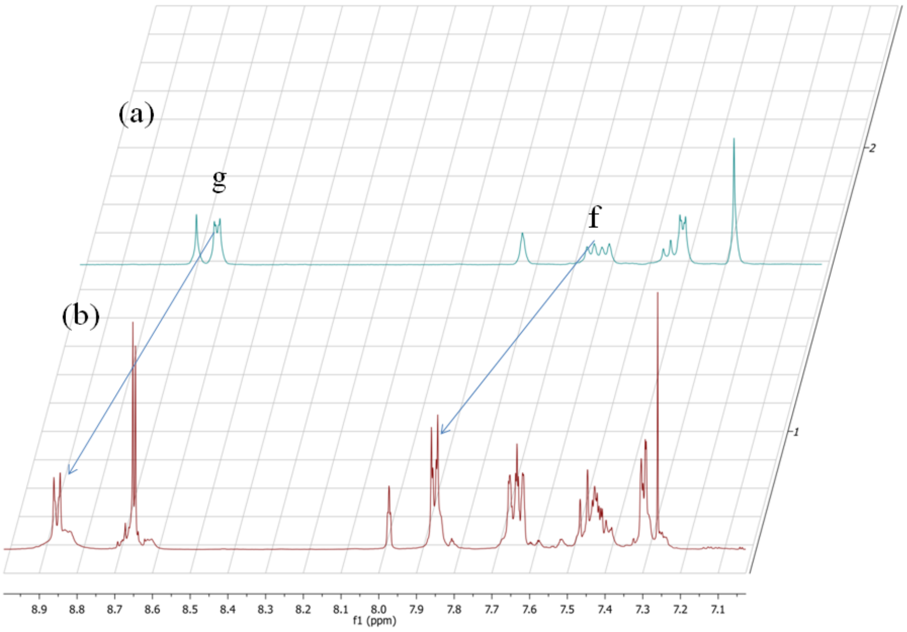

Figure S6: ^{1}H DOSY NMR spectrum of macrocycle **7**

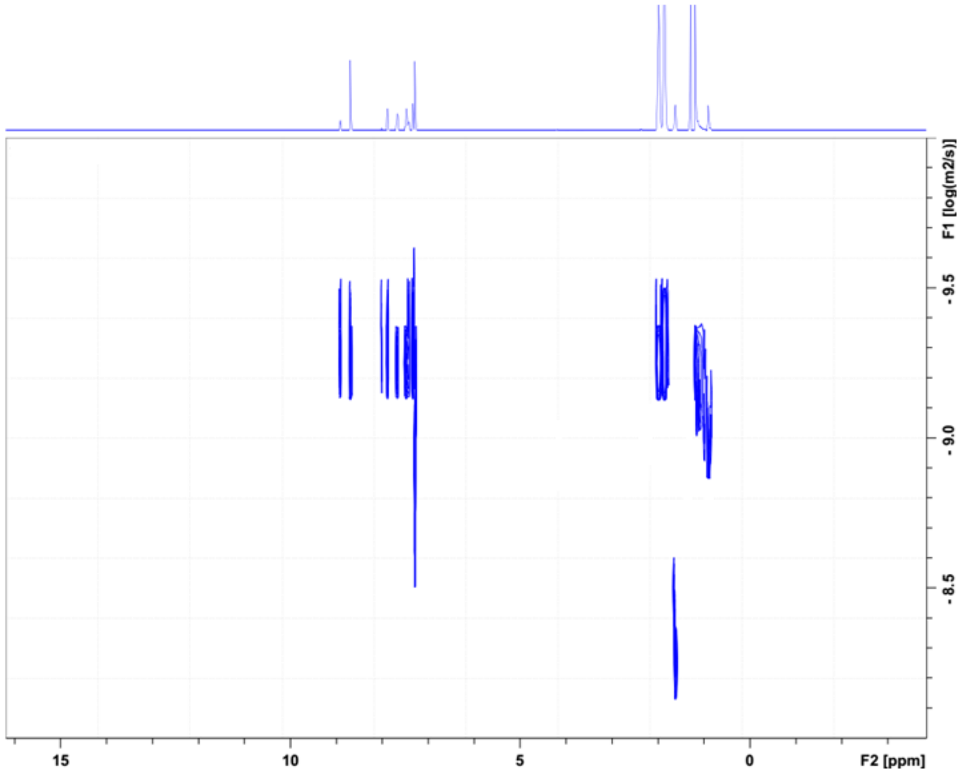

Figure S7: space-filled model of macrocycle **7** optimized by PM6 semiempirical molecular orbital method

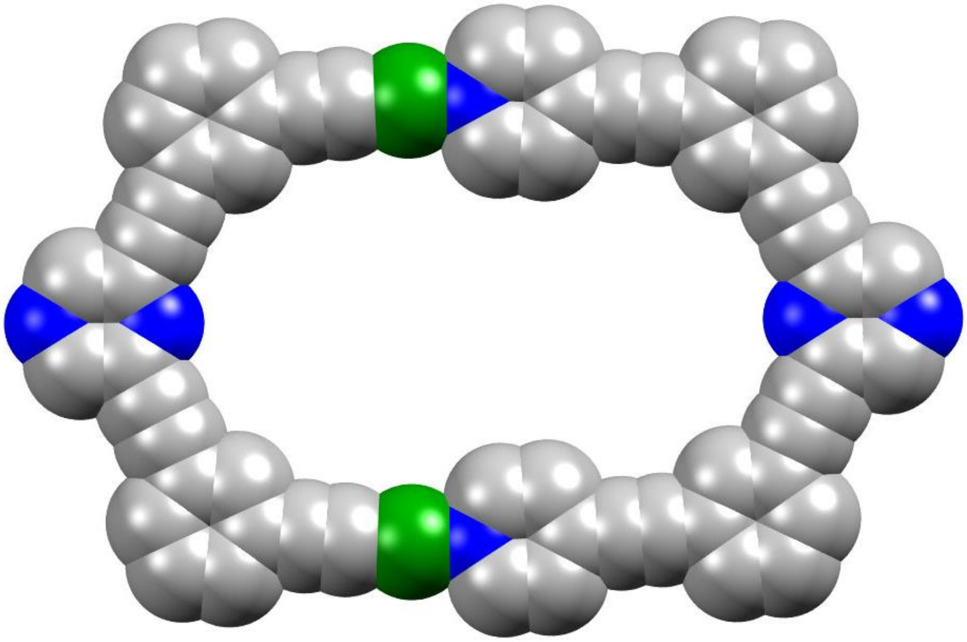

Figure S8. ESI-MS data of the macrocycle **7**; inset (a) experimental spectra and (b) theoretical isotopic distribution pattern of the fragment [**7**-2NO_3]$^{2+}$.

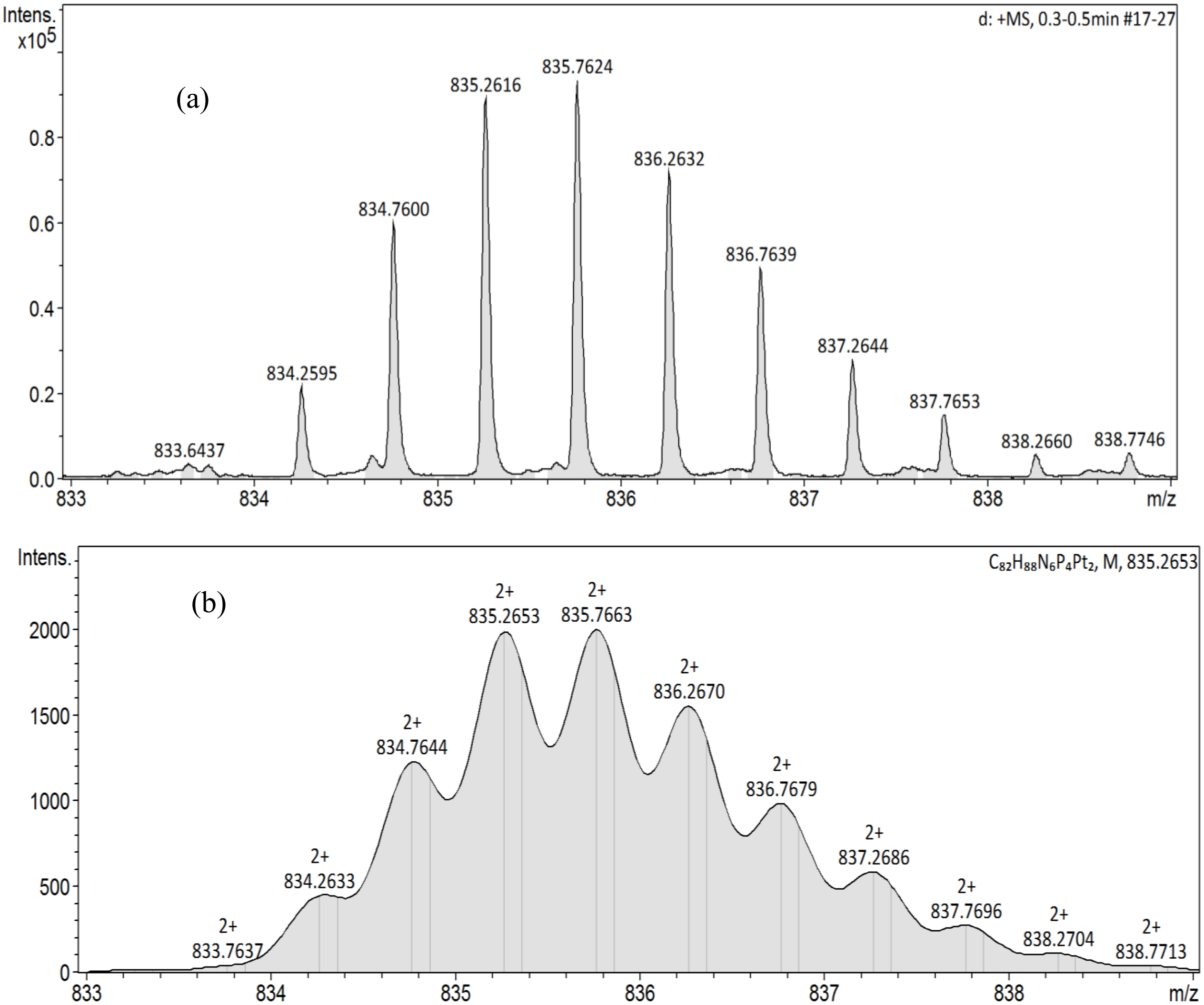

Supplement: Supplementary file 1 [file Presentation1.PDF]
